# Drug-Induced Linear IgA Bullous Dermatosis in a Critically Ill Patient Exposed to Polypharmacy

**DOI:** 10.7759/cureus.117739

**Published:** 2026-10-10

**Authors:** Sarah Bufton, Emmanuel Odega, Rabia Sarfraz, Yasmeen Al-Sadek, Sheena Goyal

**Affiliations:** 1 Dermatology, University Hospitals of Derby and Burton NHS Foundation Trust, Derby, GBR; 2 Respiratory Medicine, Kettering General Hospital, Kettering, GBR

**Keywords:** adverse drug reaction, antibacterial agents, autoimmune blistering diseases, drug-related side effects, extensive polypharmacy, intensive care units (icus), linear iga bullous dermatosis

## Abstract

Drug-induced linear IgA bullous dermatosis (DILABD) is typically associated with single-agent triggers, such as vancomycin, but its occurrence in the setting of polypharmacy is poorly understood. We report a 36-year-old female who developed DILABD during intensive care admission following exposure to multiple recognised triggering medications across different drug classes, with histological and immunofluorescence findings confirming the diagnosis. To our knowledge, this is the first reported case of DILABD arising in the context of such extensive polypharmacy, highlighting the diagnostic challenges in critically ill patients and raising the hypothesis that cumulative or synergistic drug effects may contribute to disease onset. However, this proposed interaction remains theoretical and cannot be established as a causal mechanism from a single case.

## Introduction

Linear IgA bullous dermatosis (LABD) is a rare autoimmune blistering disorder characterised by subepidermal blistering and linear deposition of IgA, with or without C3, IgG or IgM, along the dermo-epidermal junction on direct immunofluorescence [1,2]. Clinically, LABD presents with vesicles and tense bullae, which in the adult-onset form are typically on the trunk, head and limbs, and may occur in an annular ‘string of pearls’ arrangement [1]. Recognition is important as its presentation can overlap with other autoimmune and severe drug-related blistering disorders [1]. It may occur idiopathically or be drug-induced. 

Drug‑induced LABD (DILABD) is well recognised with several medications, most notably vancomycin, although the literature largely focuses on single‑agent causality [1-4]. By contrast, the role of polypharmacy, particularly involving multiple recognised triggering medications across different drug classes, remains poorly understood, with limited systematic evidence specifically examining the potential cumulative or synergistic effects of multiple DILABD-associated medications. Overlapping treatment periods and variable latency between drug exposure and rash onset can further complicate temporal assessment and attribution to a single causative agent. We report a case of DILABD arising following extensive overlapping medication exposure in the intensive care unit (ICU), highlighting the challenges of drug causality assessment and the potential contribution of combined or cumulative drug exposure to disease onset.

## Case presentation

A 36-year-old female was referred to dermatology with a blistering eruption affecting the medial arms, anterior thighs and knees, without mucosal involvement. Her past medical history included asthma, hypertension, gastroesophageal reflux disease and morbid obesity. Regular medications were Fostair (formoterol/beclometasone), salbutamol, bisoprolol, pregabalin, fluoxetine, lansoprazole and losartan. 

Approximately one month prior to dermatology review, she had been admitted to the intensive care unit (ICU) with a severe infective exacerbation of asthma secondary to community-acquired pneumonia. This was complicated by type 1 respiratory failure requiring extracorporeal membrane oxygenation (ECMO) after maximal conventional therapy was ineffective and acute kidney injury requiring renal replacement therapy. An overview of the patient’s clinical course preceding the diagnosis of drug-induced linear IgA bullous dermatosis (DILABD) is presented in Figure 1.

**Figure 1:**
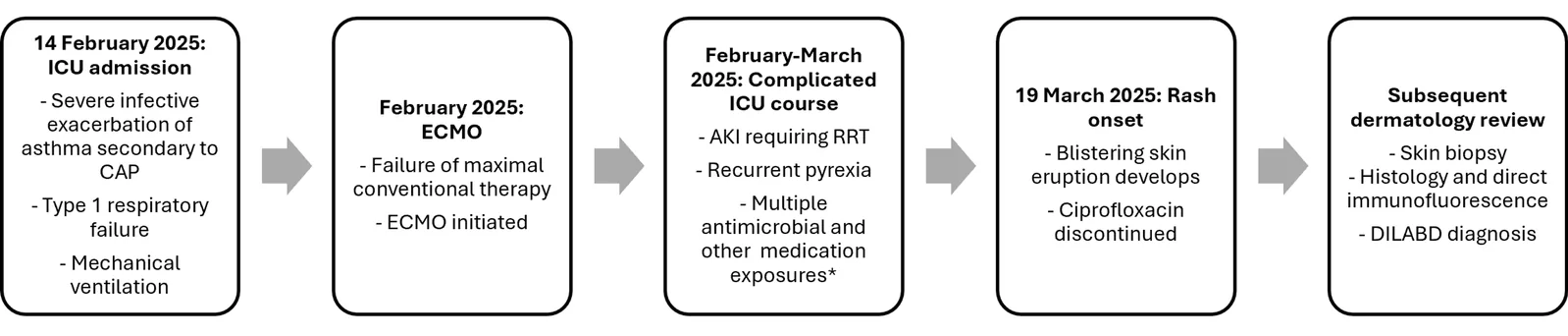
Timeline of the patient’s clinical course preceding the diagnosis of drug-induced linear IgA bullous dermatosis (DILABD). The patient’s intensive care unit (ICU) admission was complicated by respiratory failure requiring extracorporeal membrane oxygenation (ECMO), acute kidney injury (AKI) requiring renal replacement therapy (RRT), recurrent pyrexia and multiple medication exposures before the onset of a blistering skin eruption. *Detailed medication exposures and their temporal relationship to rash onset are presented in Table 1.

During admission, the patient received antimicrobial agents for pulmonary, urinary and femoral haemodialysis central line infection, resulting in unusually extensive polypharmacy. An overview of medication duration and the time from the last drug dose to the rash onset is summarised in Table 1. Medications administered known to be implicated in DILABD include levofloxacin, meropenem, vancomycin, fluconazole, amlodipine, metronidazole, paracetamol and ciprofloxacin.

**Table 1:** Medication exposures prior to the onset of drug-induced linear IgA bullous dermatosis Medication exposures prior to rash onset, showing treatment period(s), interval between the final dose and rash onset and pharmacovigilance signals reported by Yang et al. [3]. Rash onset occurred on 19/03/2025. Ciprofloxacin was discontinued on the day of rash onset due to its potential role as a trigger. * The interval represents the number of complete intervening days between the final medication dose and rash onset. † The reporting odds ratio (ROR) indicates the strength of the reporting association between a medication and LABD within the FAERS database; a higher ROR indicates a stronger reporting association. ROR does not establish causality or represent the individual risk of developing LABD. “No signal reported” indicates that no significant pharmacovigilance signal was reported for that medication by Yang et al. [3].

Medication	Treatment period(s)	Complete days between final dose and rash onset*	Pharmacovigilance signal reported by Yang et al. [3] †
Zopiclone	13/02/2025–14/02/2025	32	No signal reported
Levofloxacin	14/02/2025–16/02/2025	30	ROR 6.02 (95% CI 3.62–10.02)
Aminophylline	17/02/2025	29	No signal reported
Clarithromycin	16/02/2025–20/02/2025	26	No signal reported
Senna	02/03/2025	16	No signal reported
Clonidine	03/03/2025	15	No signal reported
Diazepam	03/03/2025	15	No signal reported
Macrogol	03/03/2025	15	No signal reported
Ipratropium bromide	05/03/2025	13	No signal reported
Fluconazole	25/02/2025–06/03/2025	12	ROR 13.07 (95% CI 6.79–25.18)
Meropenem	17/02/2025–21/02/2025; 25/02/2025–06/03/2025	12	ROR 39.67 (95% CI 21.91–71.82)
Vancomycin	25/02/2025–06/03/2025	12	ROR 692.12 (95% CI 621.74–770.46)
Erythromycin	05/03/2025–09/03/2025	9	No signal reported
Nystatin	04/03/2025–10/03/2025	8	No signal reported
Doxazosin	11/03/2025	7	No signal reported
Paracetamol	08/03/2025–11/03/2025	7	ROR 4.76 (95% CI 2.14–10.62)
Noradrenaline	11/03/2025–12/03/2025	6	No signal reported
Oxycodone	12/03/2025	6	No signal reported
Midazolam	13/03/2025	5	No signal reported
Remifentanil	12/03/2025–13/03/2025	5	No signal reported
Calcium gluconate	14/03/2025	4	No signal reported
Metronidazole	10/03/2025–15/03/2025	3	ROR 16.67 (95% CI 10.72–25.92)
Ondansetron	15/03/2025	3	No signal reported
Propofol	04/03/2025–15/03/2025	3	No signal reported
Amlodipine	14/03/2025–16/03/2025	2	ROR 11.49 (95% CI 8.79–15.01)
Teicoplanin	03/03/2025–06/03/2025; 10/03/2025–16/03/2025	2	No signal reported
Ceftazidime	13/03/2025–17/03/2025	1	No signal reported
Loperamide	13/03/2025–17/03/2025	1	No signal reported
Magnesium sulphate	15/03/2025–17/03/2025	1	No signal reported
Potassium bicarbonate	10/03/2025–17/03/2025	1	No signal reported
Ciprofloxacin	13/03/2025–19/03/2025	0 (discontinued on day of rash onset)	ROR 6.51 (95% CI 4.48–9.47)

On examination, tense bullae on an erythematous base were arranged in a characteristic string-of-pearls distribution on the medial arms, anterior thighs and knees (Figure 2A-2C). There was no mucosal involvement. Skin biopsy demonstrated subepidermal blistering with a predominantly neutrophilic infiltrate with occasional eosinophils (Figure 3A). Direct immunofluorescence showed linear deposition of IgA and C3 along the basement membrane zone (Figures 3B, 3C), consistent with DILABD.

**Figure 2:**
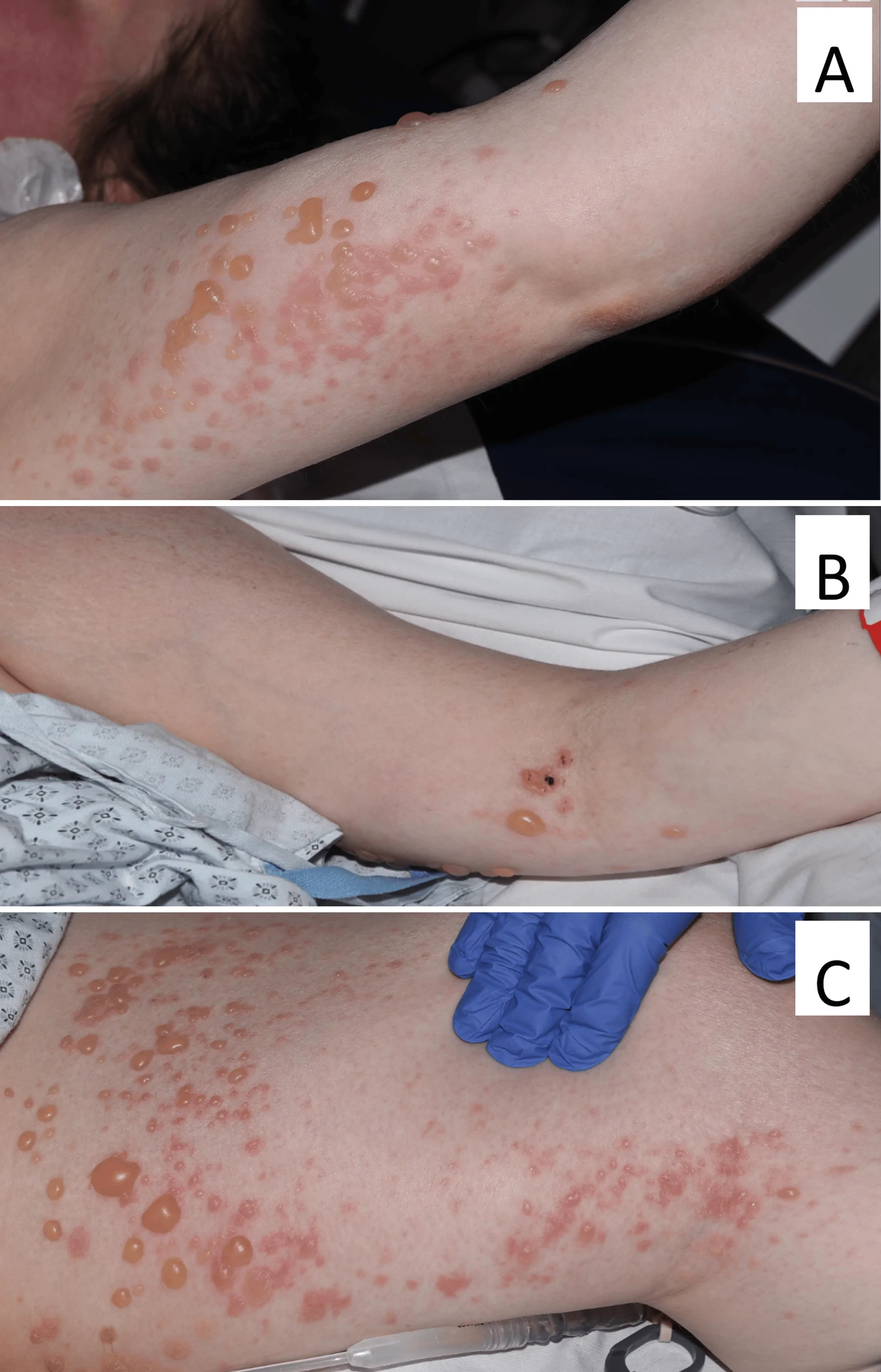
Tense bullae on an erythematous base involving the medial arm (A), elbow (B) and medial thigh and knee (C).

**Figure 3:**
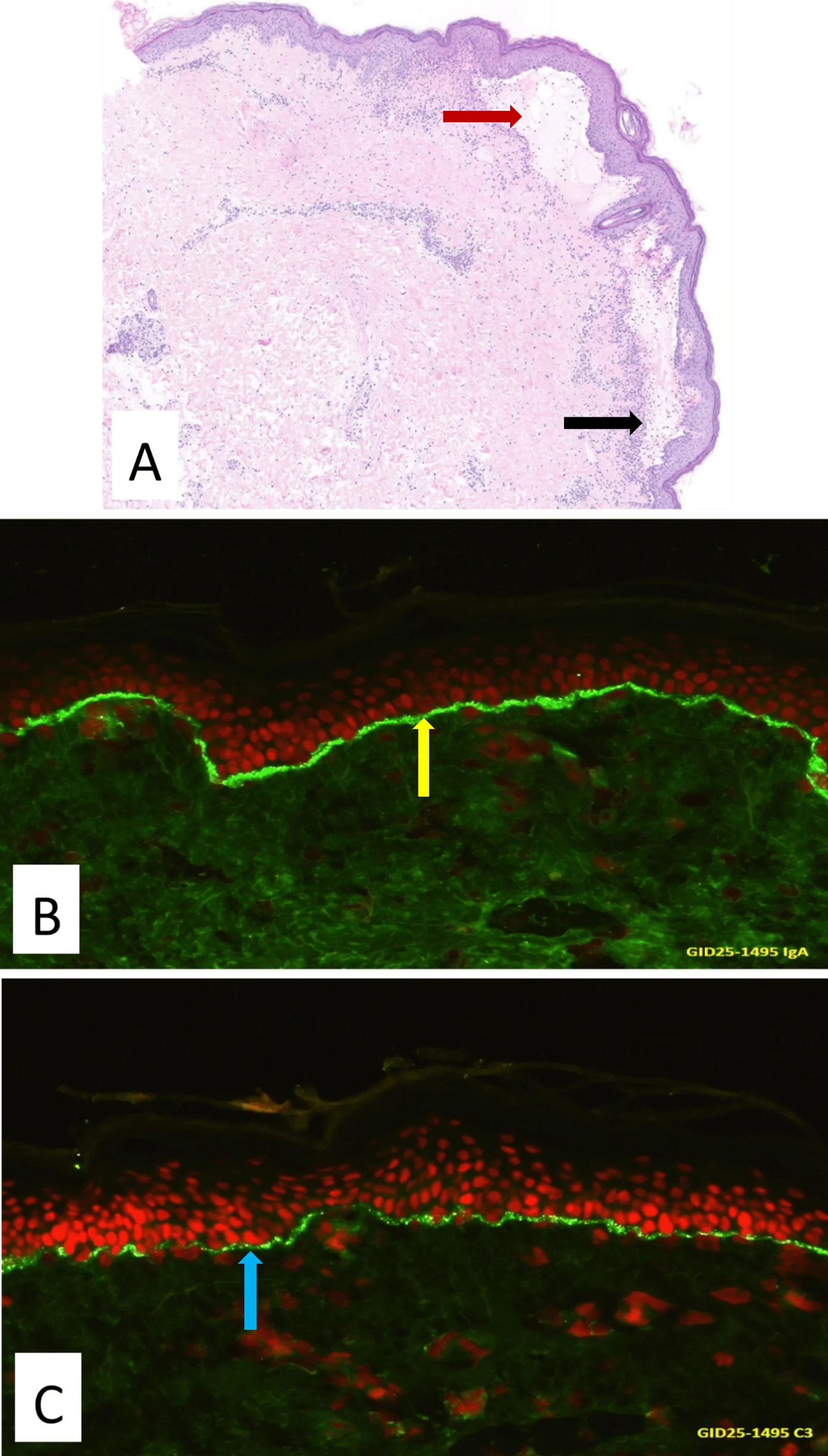
Histology findings following skin biopsy. Histology specimen (A) demonstrates subepidermal blistering (red arrow) with a mixed inflammatory infiltrate (black arrow) with neutrophil-dominance and occasional eosinophils. Direct immunofluorescence images show linear deposition of IgA (B) and C3 (C) along the basement membrane zone, as indicated by the yellow and blue arrows, respectively.

In this case, the overlapping exposure to several potential culprit drugs made causality assessment particularly complex, especially where ongoing antimicrobial therapy was required. As other potential trigger agents had already been discontinued prior to rash onset, ciprofloxacin was stopped as it was the only potentially implicated triggering medication still being administered at the time. Antimicrobial therapy was rationalised, and the patient was managed topically with clobetasol propionate following blister lancing, with subsequent improvement. At the eight- and 12-week follow-up post-discharge and to date, there has been no recurrence, supporting a DILABD diagnosis. 

## Discussion

Idiopathic LABD has been associated with malignancy, infections, autoimmune disease and inflammatory bowel disease [1,2]. DILABD presents similarly, including vesicles, tense bullae and the string-of-pearls sign, but may be more extensive and severe and can resemble toxic epidermal necrolysis, necessitating prompt recognition and management [1,2]. 

Disease severity and distribution in DILABD are not shown to correlate with serum drug levels, limiting the utility of therapeutic drug monitoring in establishing causality [2]. Furthermore, there are no histological or immunological markers that reliably distinguish idiopathic LABD from DILABD [1]. Both forms share common target antigens, including LAD285 and BP180, with indistinguishable direct immunofluorescence findings and non-specific histology demonstrating subepidermal blistering with inflammatory infiltrate [1]. Diagnosis therefore relies heavily on clinical context, drug history and temporal relationships between drug exposure and disease onset, which become particularly complex in the setting of extensive polypharmacy, as in this case. 

Vancomycin is the most frequently implicated causative agent in DILABD and carries the strongest signal in pharmacovigilance studies [3,4]. Other agents reported as triggers include trimethoprim‑sulfamethoxazole, metronidazole, ciprofloxacin, meropenem, fluconazole, paracetamol, penicillins, cephalosporins, anti-hypertensives, immunosuppressants, anti-epileptics, checkpoint inhibitors and amlodipine [1,3-8]. The latency period between drug exposure and onset of cutaneous symptoms is highly variable, ranging from 24 hours to several weeks and, in rare cases, exceeding 780 days [1,4]. In our case, all potential culprit medications were administered within this window; however, most had been discontinued prior to rash onset. Rechallenge testing carries risk of severe recurrence and is rarely justified in clinical practice; it was not performed in this case [1]. 

The temporal relationships of the medication exposures and pharmacovigilance signals reported by Yang et al. [3] are summarised in Table 1. The pattern of overlapping exposure to multiple potential triggering medications is central to the diagnostic challenge in this case. Although vancomycin represents the most likely single-agent trigger based on existing evidence, the absence of a clear temporal relationship, combined with extensive concurrent drug exposure, precludes confident attribution to a single agent. This exposure pattern raises the hypothesis that cumulative or synergistic effects may contribute to DILABD onset; however, this case cannot establish a causal relationship or confirm that such an interaction occurred.

Although several of the medications administered have been individually associated with DILABD [1,3,4], the literature does not address whether sequential or concurrent exposure produces additive or synergistic immunological effects. Mechanisms of drug-induced anti-IgA autoimmunity remain poorly understood, although polypharmacy is a recognised confounder in drug causality assessment [3,4]. 

Theoretically, concurrent drug exposure may promote immune dysregulation via cumulative antigenic load, epitope spreading or cytokine-mediated pathway amplification, potentially lowering the threshold for autoantibody formation against basement membrane antigens [1]. In this context, the development of DILABD in the setting of extensive polypharmacy, as observed in our case, may reflect complex, as yet unelucidated immunological interactions rather than a single-drug effect. 

In addition, establishing causality in DILABD is particularly challenging in critically ill patients. While polypharmacy is common in intensive care settings, the breadth, duration and overlap of antimicrobial exposure in this case were unusually extensive. Critically ill patients also exhibit a uniquely complex pharmacokinetic environment due to severe physiological alterations: increased volume of distribution, variable renal and hepatic clearance, inflammation, and the effects of life-support therapies such as ECMO and continuous renal replacement therapy (CRRT) [9]. 

Thus, in this case, the concurrent use of multiple renally excreted medications in acute kidney injury may have led to drug accumulation and enhanced immune activation, potentially contributing to DILABD development [3,5]. A clearer understanding of drug-specific risk and underlying mechanisms would be vital for identifying high-risk patients, informing prescribing decisions in critically ill populations, and developing monitoring strategies to mitigate severe disease. 

## Conclusions

This case highlights the diagnostic and therapeutic challenges of DILABD in the setting of ICU polypharmacy, where withdrawal of all potential trigger medications is often not feasible. The delayed onset and extensive overlap of medication exposures precluded attribution to a single agent. While synergistic or cumulative effects remain unproven, the exposure pattern in this case, supported by plausible immunological mechanisms, raises the hypothesis that polypharmacy may contribute to DILABD onset in susceptible individuals; however, such an effect cannot be established from a single case. Systematic reporting of similar cases is needed to clarify the impact of concurrent drug exposure on DILABD pathogenesis. Early recognition and multidisciplinary management remain essential in critically ill patients presenting with blistering eruptions.
